# Beliefs and self-reported practice of footcare among persons with type II diabetes mellitus attending selected health centres in east Trinidad

**DOI:** 10.1186/s43162-022-00180-2

**Published:** 2022-12-17

**Authors:** Rachel Leah Vincent-Edinboro, Philip Onuoha

**Affiliations:** The UWI School of Nursing, Faculty of Medical Sciences, University of the West Indies, St. Augustine, Trinidad, Trinidad and Tobago

**Keywords:** Beliefs, Practices, Diabetes, Footcare, Caribbean

## Abstract

**Background:**

It has been documented that nearly 600 million people worldwide are expected to have diabetes mellitus in 2035 and that approximately 140,000 persons aged 20–69 years living with diabetes mellitus in Trinidad and Tobago. It is also indicated that patients with type II diabetes mellitus face a higher risk of diabetic foot neuropathy and foot ulcers which increase the risk of below the knee amputation in persons living with diabetes.

**Purpose:**

The aim of this research project was to explore the beliefs related to footcare and the self-reported footcare practice of persons with type II diabetes mellitus attending selected health centres in East Trinidad.

**Method:**

A survey was used to explore the footcare beliefs and the self-reported footcare practice of persons with type II diabetes mellitus attending selected health centres in East Trinidad (*n* = 87).

**Results:**

Participants had strong belief regarding susceptibility to foot injury, strong belief regarding the seriousness of complications of foot injury, reported good footcare practice and excellent practice of overcoming barriers to performing footcare. There is a correlation between the belief regarding susceptibility to a foot injury and age (*p* ≤ 0.05). Also, there is a significant correlation between belief regarding susceptibility to a foot injury, seriousness of complications and self-reported footcare practices (*p* ≤ 0.05).

**Conclusion:**

This study explores and describes the beliefs and self-reported practices of footcare among individuals with type II diabetes mellitus at selected health centres in East Trinidad. It supports the Health Belief Model as an effective framework for the promotion of appropriate footcare among persons with type II diabetes mellitus.

## Introduction

Nearly 600 million people worldwide are expected to have diabetes mellitus by 2035 [1]. There is approximately 140,000 persons aged 20–69 years living with diabetes mellitus in Trinidad and Tobago [1]. Patients with type II diabetes mellitus can develop diabetic foot neuropathy which increases the risk of injuring the feet and developing potentially fatal complications [2]. Other diabetic foot complications include ulceration, Charcot foot, gangrene and amputation [3]. Foot ulcers increase the risk of below the knee amputation in persons living with diabetes. One lower limb is lost every 30 s around the world due to diabetic foot ulceration [4]. Trinidad and Tobago performs approximately 500 limb amputations annually [5]. The treatment of diabetic foot is very lengthy and also very expensive [4]. Diabetic foot problems are one of the most costly, disabling and disheartening complications of diabetes mellitus [4]. Treatment of patients with diabetes places a substantial burden on the national expenditure, accounting for 5.21% of the gross domestic product in Trinidad and Tobago or an estimated US $467 million [6]. Treatment of foot infections accounts for 29% of the expenditure, as it is the most common complication of diabetes requiring hospital admission and it accounts for 14% of all hospital admissions in Trinidad and Tobago [5]. Certainly, diabetic foot ulceration poses a heavy burden on the patient and the healthcare system, and the prevention thereof deserves more attention [1]. Self-care activities are the cornerstone of diabetes care that ensures patients actively contribute to the prevention of complications. As such, the beliefs regarding footcare among diabetic patients are receiving attention as they are likely to influence the footcare practice of individuals.

Beliefs are a fundamental and significant aspect of human cognition that fulfil important individual and social functions [7]. According to Connors and Halligan [8] ‘belief can be defined as mental acceptance or conviction in truth or actuality of some idea.’ Thus, the two properties of belief are representational content and assumed veracity [8]. Fuentes [9] proposed that belief is the capacity to draw on a range of cognitive and social resources, histories, and experiences. Although beliefs can remain unconscious or outside of immediate awareness, they offer a moral compass, guide our relationships, motivate behaviours in both large and small affairs and provide meaning and comfort [7, 8, 10–12].

This phenomenon was evident in a study from Papua New Guinea which found that perceptions and beliefs about the cause of foot ulcers, particularly the ‘belief in sorcery’ played a role in behaviours related to footcare and was the most common cause of late presentation of diabetic septic foot [13]. Literature suggests that individuals cannot arbitrarily choose to believe anything or change a belief on a personal whim, and knowing that an individual has a true belief about some situation may not allow for the prediction of actions if the attributed beliefs are underspecified [9, 14].

If proper footcare is not done by persons with diabetes, then certain complications of diabetes mellitus especially diabetic foot ulcers can occur [15]. The lifetime risk of a person with diabetes developing a foot ulcer could be as high as 25% [16, 17]. Diabetic peripheral neuropathy is the most common cause of neuropathy worldwide, and its prevalence increases with the duration of diabetes [18]. Literature shows that diabetic peripheral neuropathy affects 50–60% of diabetic patients [18, 19]

The International Diabetes Federation (IDF) screening chart classifies the extent of risk an individual with diabetes may experience as either ‘low risk,’ ‘moderate risk,’ ‘high risk’ or ‘very high risk’ [20]. According to the IDF screening chart, an individual with normal plantar sensation will be considered to be at ‘low risk,’ and an individual who has loss of protective sensation is considered to be at ‘moderate risk’ [21]. The IDF screening chart states that an individual who has loss of protective sensation with either higher pressure, poor circulation, structural foot deformities or onychomycosis is considered to be at ‘high risk’ and an individual with a history of ulceration, amputation or neuropathic fracture is considered to be at ‘very high risk’ [20].

Practice is the act of doing or performing some skill often, customarily or habitually [22]. When individuals learn a new skill, even with clear instructions, it is often necessary to practice for hours, weeks or months before the achievement of proficient and fluid performance [23]. The effects of practice on behaviour, including increasing the speed of performance, rendering the practised behaviour habitual and reducing the cognitive load required to perform the task [24]. Despite motor skills advance with repetition, repeated practice will be of minimal benefit if the individual is not given feedback on the performance, indicating whether the practice is right or incorrect [24]. All patients, if given proper guidance and education regarding footcare, should be able to improve their footcare practice [25].

Footcare knowledge help with preventing foot ulcers in diabetic patients [1, 11, 13, 26]. However, evidence shows that people with diabetes mellitus often fail to employ the behavioural strategies suggested in educational interventions and discrepancies in footcare practice persist despite understanding appropriate footcare behaviours [6, 17, 26, 27]. Reasons for none adherence are complex, considering behaviour has often been conceptualized as a function of environmental, personal and biological factors [13, 27].

The American Diabetes Association states that ‘people with diabetes should perform a comprehensive foot evaluation at least annually to identify risk factors for ulcers and amputations.’ Components of the foot screening process involve palpation of the pedal pulses, a sensory examination to determine protection sensation and inspection for foot deformities [22]. While health care providers are responsible for performing an annual comprehensive foot examination, individuals with diabetes are expected to perform self-footcare to prevent injury to feet and subsequent complications [22]. Good footcare behaviours include daily inspection of feet for problems such as colour change, swelling, breaks in the skin, pain or numbness. Guidelines specific to appropriate footcare practice emphasize hygiene, such as daily washing and carefully drying the feet and nail care, as well as wearing well-fitting shoes and hosiery to help minimize the risk of foot complications [20, 28].

If an individual with diabetes notices red sore spots, blisters, corns, calluses or pain associated with wearing shoes, then new properly fitting footwear should be obtained as soon as possible [29]. Yet, a systematic review of literature related to footcare knowledge and self-footcare practice found that ‘many patients did not inspect their feet regularly or inspect the insides of their shoes.’ [20]. More recent evidence, pointed out 21 out of 106 persons with diabetes mellitus were not sure after washing feet if they had to dry the feet in between toes and were unaware that they should cut their nails straight rather than follow the shape of the nails [24]. Cutting toenails with sharp instruments such as razor blades is considered an improper footcare practice because it increases the risk of injury to the foot. Diabetic patients generally have a mixture of proper and improper footcare practices [18].

Uncontrolled diabetes can cause visual disturbance, and an alteration in visual acuity can be a challenge to self-examination or monitoring feet [22]. Persons with visual difficulties, physical constraints preventing movement or cognitive problems that impair the ability to assess the condition of the foot and institute appropriate responses will need other people such as a family member to assist with their care. Physical limitations of an individual with diabetes to perform self-examination or monitor feet can be overcome with the use of mirrors [22]. Zhong et al. [30] suggest that a good pedicure treatment may lower the risk of getting diabetic foot ulcers. However, the possibility of not feeling pain if nicked or cut during a pedicure could cause more harm than good. Thus, to avoid the negative impact of bad podiatric conditions in diabetic patients, pedicures must be performed by professionals [30].

**Fig. 1:**
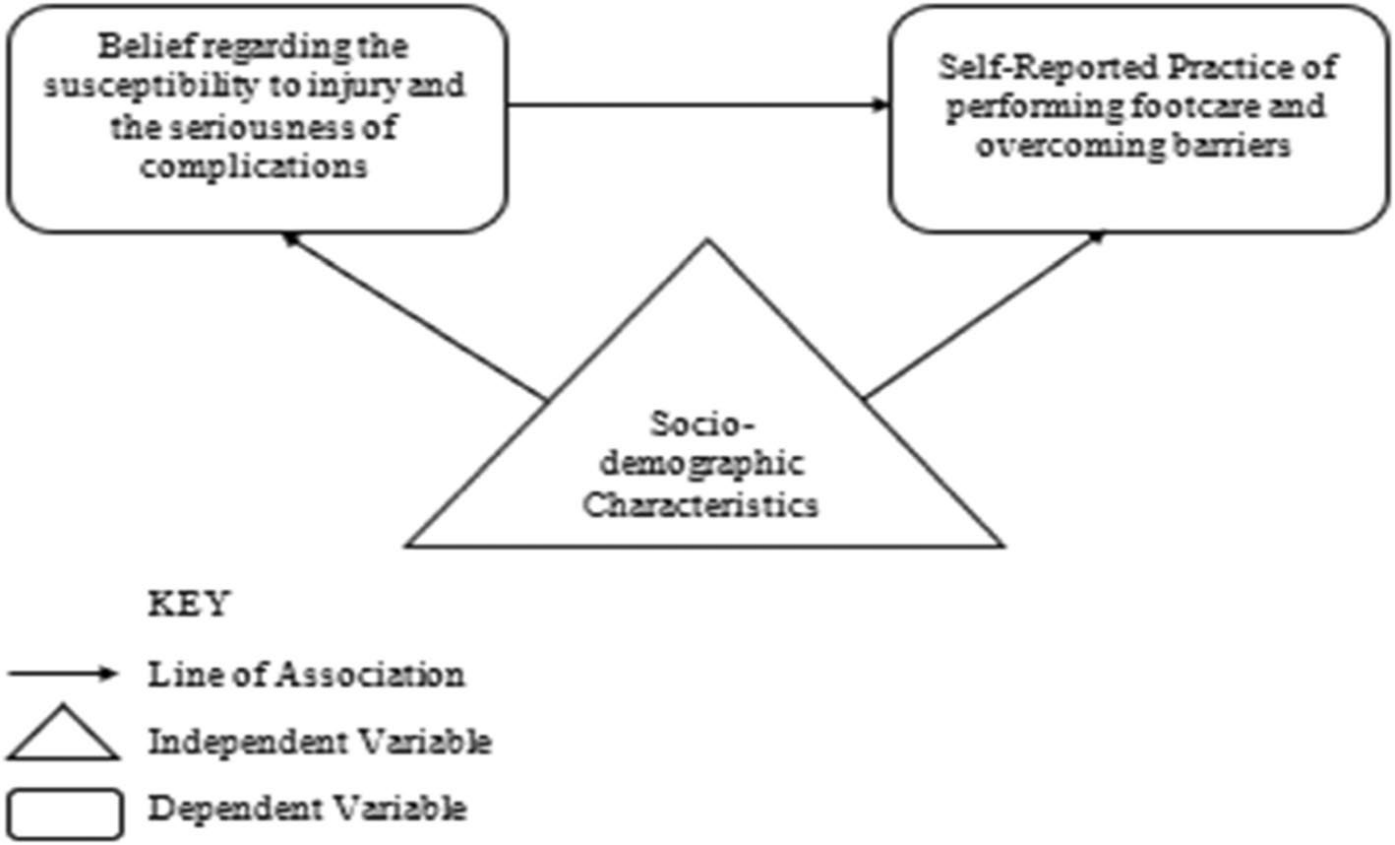
A modified Health Belief Model for diabetic footcare

**Fig. 2:**
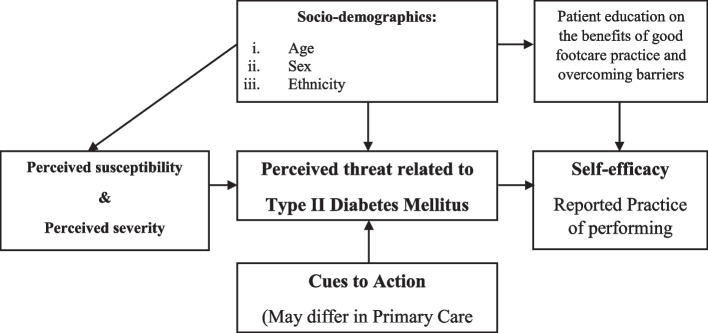
The association between the independent and dependent variables

In this study, we have sought to explore the Health Belief Model (HBM) developed in the 1950s to describe why some people who are free of illness will take actions to prevent illness, whereas others fail to do so [31]. To reduce the complications of diabetes, some studies emphasize that healthcare workers should not merely provide knowledge to people but consider the perception of the risk as a central concept for understanding healthy behaviours and making changes in behaviour [20]. Evidence has shown that individuals will take action if two conditions are present: (i) a perceived threat to personal health and (ii) the conviction that the benefits of taking action to protect health outweigh the barriers that will be encountered [20]. Thus, the perceived threat to personal health related to type II diabetes mellitus is specific to foot injury and complication. The individual perceptions that will be explored are the beliefs regarding susceptibility to a foot injury and the beliefs regarding the severity of complications of injury to feet. The conviction that leads an individual to act to protect the feet comes from knowledge about appropriate footcare practice and ways to overcome the barriers to performing footcare. Cues to action and patient education strategy may differ in each primary care setting. Yet, the sociodemographic characteristics of individuals as well as the cues to action can also influence the perceived threat of foot injury and foot self-care practice. The concepts of the HBM have been modified in the theoretical framework as illustrated in Fig. 1. We conceptualize therefore that the demographic characteristics of the participants would be related to their beliefs as well as their reported practice as illustrated in Fig. 2 which are the relationships between the dependent and the independent variables of the study.

### Aim

The specific aim of this research project is to explore the footcare beliefs and the self-reported footcare practice of persons with type II diabetes mellitus attending selected health centre sin East Trinidad.

### Research questions

1. What are the sociodemographic characteristics of persons with type II diabetes mellitus attending selected health centres in East Trinidad?
2. What are the beliefs of persons with type II diabetes mellitus attending selected health centres in East Trinidad regarding:

i. The susceptibility to foot injury?
ii. The seriousness of complications of injury to the foot?

3. What is the self-reported practice of persons with type II diabetes mellitus attending selected health centres in East Trinidad regarding:

i. Performing footcare?
ii. Overcoming barriers to performing footcare?

4. Are there any associations between:

i. The sociodemographic characteristics and the beliefs of persons with type II diabetes mellitus attending selected health centres in East Trinidad?
ii. The sociodemographic characteristics and the self-reported practice of persons with type II diabetes mellitus attending selected health centres in East Trinidad?

5. Are there any associations between the belief:
  i. Related to the susceptibility to a foot injury
  ii. Regarding the seriousness of complications of injury to the foot, and the self-reported practice:

- Performing footcare
- Overcoming barriers to performing footcare, of persons with type II diabetes mellitus attending selected health centres in East Trinidad?

### Methodology

This is a quantitative, cross-sectional and descriptive study of the beliefs and self-reported practice of footcare among persons with type II diabetes in East Trinidad.

**Fig. 3:**
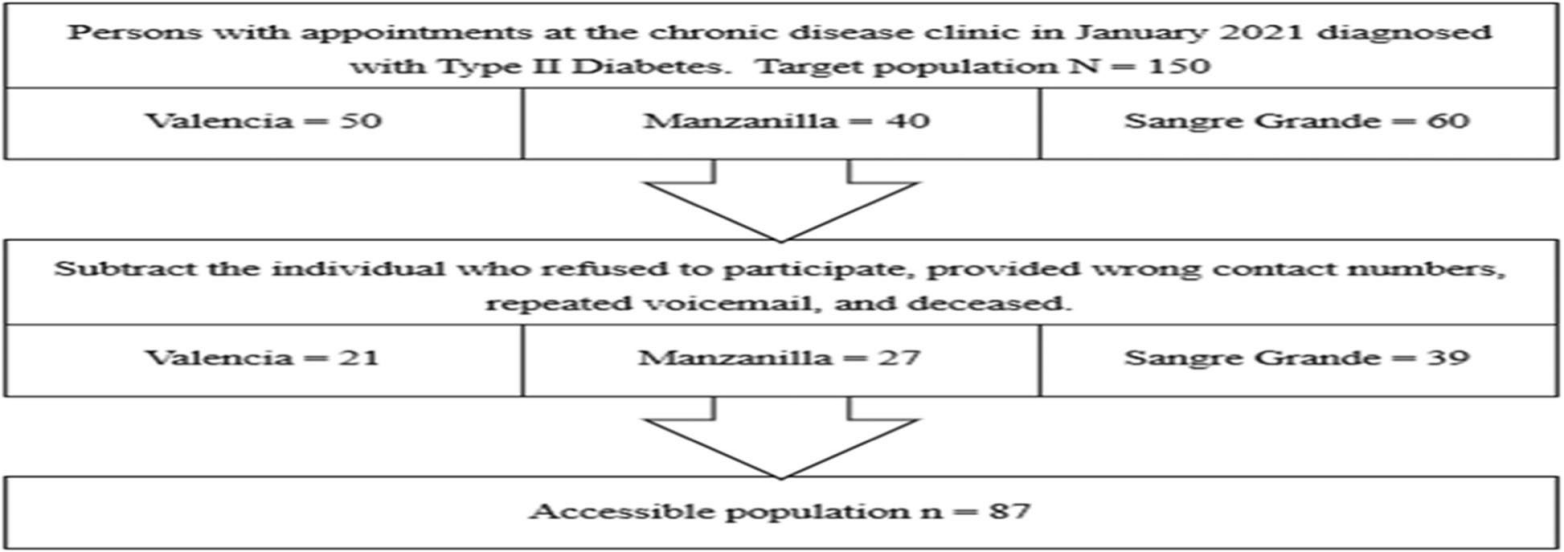
Segmented process of obtaining the sample population

Persons diagnosed with type II diabetes mellitus in East Trinidad and attending chronic disease clinic at selected health centres within the ERHA were targeted. We recruited all the individuals with appointments for January 2021 at the chronic disease clinics of three (3) selected health centres within the ERHA: Sangre Grande Enhanced Health Facility, Manzanilla Outreach Facility, and Valencia Outreach Facility. Based on our knowledge, the selected health centres were the main chronic disease clinics that served the largest patient population of the region. Individuals that met the inclusion criteria of the study were shortlisted and contacted. A total of 150 persons were enlisted. However, 87 were assessed as represented in Fig. 3.

The following were the inclusion criteria for the study:

- Adult persons (> 18 years age) with the diagnosis of diabetes mellitus II attending chronic disease clinics at a health centre within ERHA
- Persons who give consent to participate in the study
- Provided a functional contact number to the health centre

The study was carried out in a virtual space, as the participants were contacted via telephone between the hours 8 am and 4 pm, Monday to Friday, in January 2021. This was at the time of COVID-19 lockdown in the country of Trinidad and Tobago.

#### Ethical consideration

Ethical approvals were sought and obtained from the Campus Ethics committee of the University of the West Indies, St. Augustine, Trinidad and Tobago, and the Ethics Committee of the Eastern Regional Health Authority Research Committee, Trinidad and Tobago. The reference numbers are Ref: CRES-SA.0373/05/2020 and PHO: 35/20: ERHA-REC.021/09/2020 respectively. Privacy and confidentiality were maintained throughout the period of the study, and we believe that the questions items in the questionnaire were not unnecessarily intrusive.

#### Selection and development of instruments

Telephone questionnaire was designed by the researchers. The instrument was guided by literature for this study. The structure of the questionnaire utilizes concepts from the Health Belief Model to explore the beliefs and self-reported practice of footcare among persons with type II diabetes mellitus attending selected health centres in East Trinidad.

The instrument consists of 34 items divided into five (5) sections:

Section 1—Sociodemographics (8 items)

Section 2—Susceptibility to a foot injury (5items)

Section 3—Seriousness of complications (5 items)

Section 4—Performance of footcare (10 items)

Section 5—Overcoming barriers to performing footcare (6 items)

The instrument has a Cronbach’s alpha of 0.881.

#### Procedure for data collection

Individuals that met the inclusion criteria were contacted via telephone between the hours 8 am and 4 pm, Monday to Friday. Full disclosure was given about the research project, verbal consent was obtained, and the questionnaire was completed via telephone interview.

#### Data analysis

Data was manually collected and inputted into IBM SPSS Statistics Version 22. Section 2, section 3, and section 4 of the questionnaire were scored using a 5-point system. The most correct responses were given a score of ‘5’ decreasing in value to the least correct response given a score of ‘1.’ The summation of the scores from section 2 and section 3 was tabulated in groups with the following ranges: 20–25 (strong belief), 11–19 (neutral belief) and 10 or less (weak belief). The summation of the scores from section 4 was tabulated in groups with the following ranges: 40–50 (good practice), 21–39 (fair practice) and 20 or less (poor practice). Section 5 items were scored, and the percentage of correct responses were tabulated in groups with the following ranges: 100% (excellent practice), 80% (good practice) and 60% or less (poor practice). The categorization of scores is illustrated in Table 1. Descriptive and inferential statistics such as frequency and ANOVA (analysis of variance) were done to answer the research questions.

**Table 1:** Categorization of scores

*Dependent variable*	*Score*	*Meaning*
*Belief regarding susceptibility to a foot injury* *(Section 2)*	*20*–*25* *11*–*19* *10 or less*	*Strong belief* *Neutral belief* *Weak belief*
*Belief regarding the seriousness of complications of foot injury* *(Section 3)*	*20*–*25* *11*–*19* *10 or less*	*Strong belief* *Neutral belief* *Weak belief*
*Self-reported practice of performing footcare* *(Section 4)*	*40*–*50* *21*–*39* *20 or less*	*Good practice* *Fair practice* *Poor practice*
*Self-reported practice of overcoming barriers to performing footcare* *(Section 5)*	*100%* *80%* *60% or less*	*Excellent practice* *Good practice* *Poor practice*

## Results

The results of the study are presented according the research question.

To answer research question 1, *What are the sociodemographic characteristics of persons with type II diabetes mellitus attending selected health centres in East Trinidad?*, 87 persons consented and participated in the telephone interview to complete questionnaires. The response rate was 58%. The participants attended the following health centres in East Trinidad: Sangre Grande 39 (44.8%), Manzanilla 27 (31.0%) and Valencia 21 (24.1%). In response to question 1, the sociodemographic characteristics of persons with type II diabetes mellitus attending selected health centres in East Trinidad are presented in Table 2. As can be observed, the participants were mostly females (62.1%), age range 49–65 (57.5) and Christians (75.9%). Indians constituted 44.8% of the participants, with secondary school level education being the highest educational level attained by majority of the participants (63.2%). With regard to the employment status of the respondents, 23.0% of them were unemployed, and 39.1% of them are retired. Most of the (67.8%) have been diagnosed type 11 diabetes for more than 5 years.

**Table 2:** Sociodemographic characteristics of persons with type II diabetes mellitus attending selected health centres in East Trinidad (*n* = 87)

*Demographics*	*Frequency (%)*
*Gender*	
*Male*	*33 (37.9)*
*Female*	*54 (62.1)*
*Age*	
*18 to less than 29*	*0 (0)*
*29 to less than 49*	*15 (17.2)*
*49 to less than 65*	*50 (57.5)*
*65 and above*	*22 (25.3)*
*Ethnicity*	
*African*	*27 (31.0)*
*Indian*	*39 (44.8)*
*Mixed*	*21 (24.1)*
*Other*	*0 (0)*
*Religion*	
*Christian*	*66 (75.9)*
*Hindu*	*13 (14.9)*
*Muslim*	*2 (2.3)*
*Other*	*6 (6.9)*
*Educational level*	
*Primary*	*13 (14.9)*
*Secondary*	*55 (63.2)*
*Tertiary*	*19 (21.8)*
*Other*	*0 (0)*
*Employment status*	
*Employed*	*33 (37.9)*
*Unemployed*	*20 (23.0)*
*Retired*	*34 (39.1)*
*Length of time since diagnosis of type II diabetes mellitus*	
*Less than 1 year*	*10 (11.5)*
*2*–*5 years*	*18 (20.7)*
*More than 5 years*	*59 (67.8)*
*Health centre*	
*Sangre Grande*	*39 (44.8)*
*Manzanilla*	*27 (31.0)*
*Valencia*	*21 (24.1)*

**Fig. 4:**
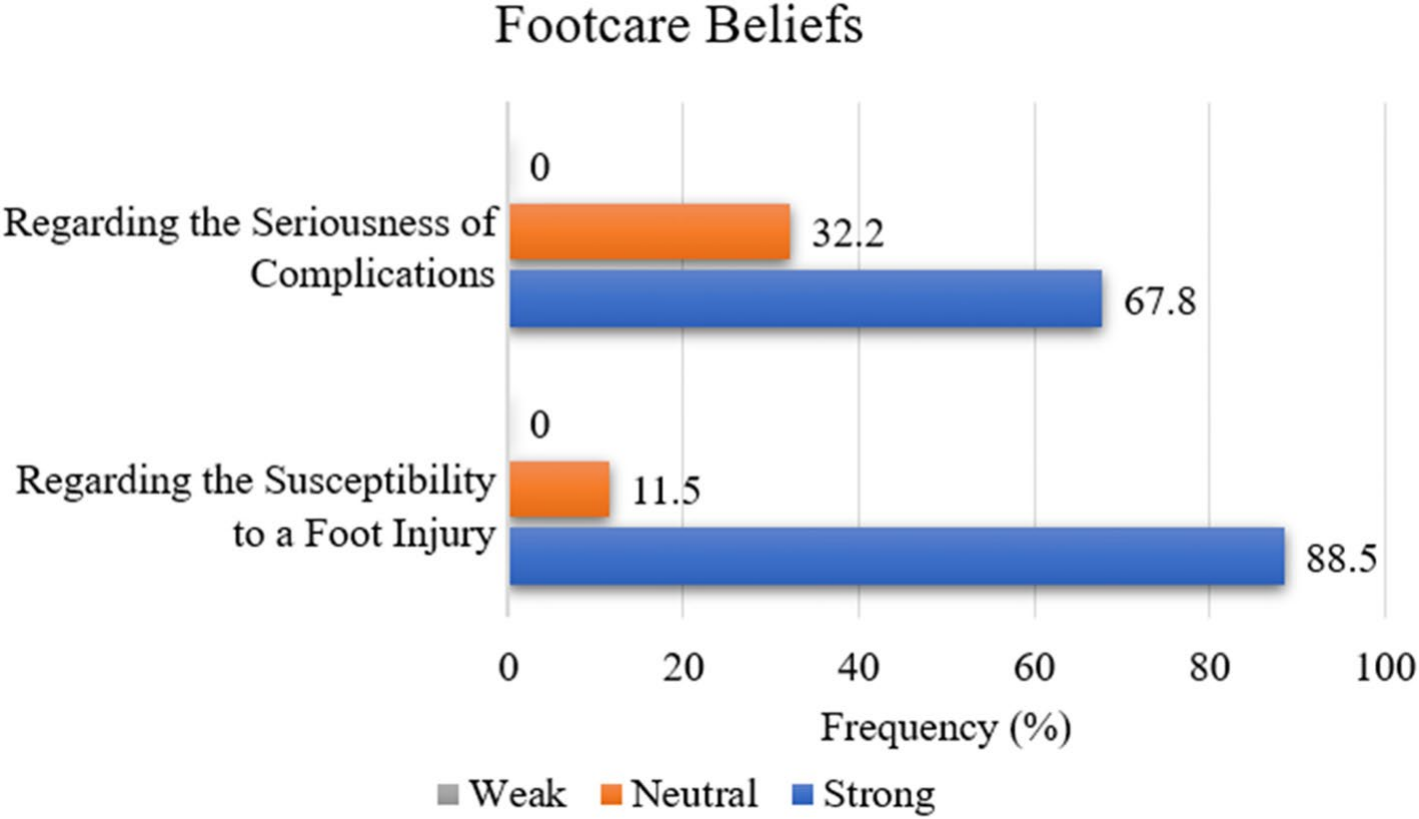
Distribution of footcare beliefs (*n* = 87)

To answer research question 2, w*hat are the beliefs of persons with type II diabetes mellitus attending selected health centres in East Trinidad regarding; i. the susceptibility to a foot injury and ii. the seriousness of complications of injury to the foot?*, the scores of participants were used to determine the beliefs regarding the susceptibility to a foot injury and their belief regarding the seriousness of complications. The distribution of scores from section 2 of the questionnaire (belief regarding the susceptibility of a foot injury) is illustrated in Fig. 4. Most of the participants (88.5%) had strong belief.

**Fig. 5:**
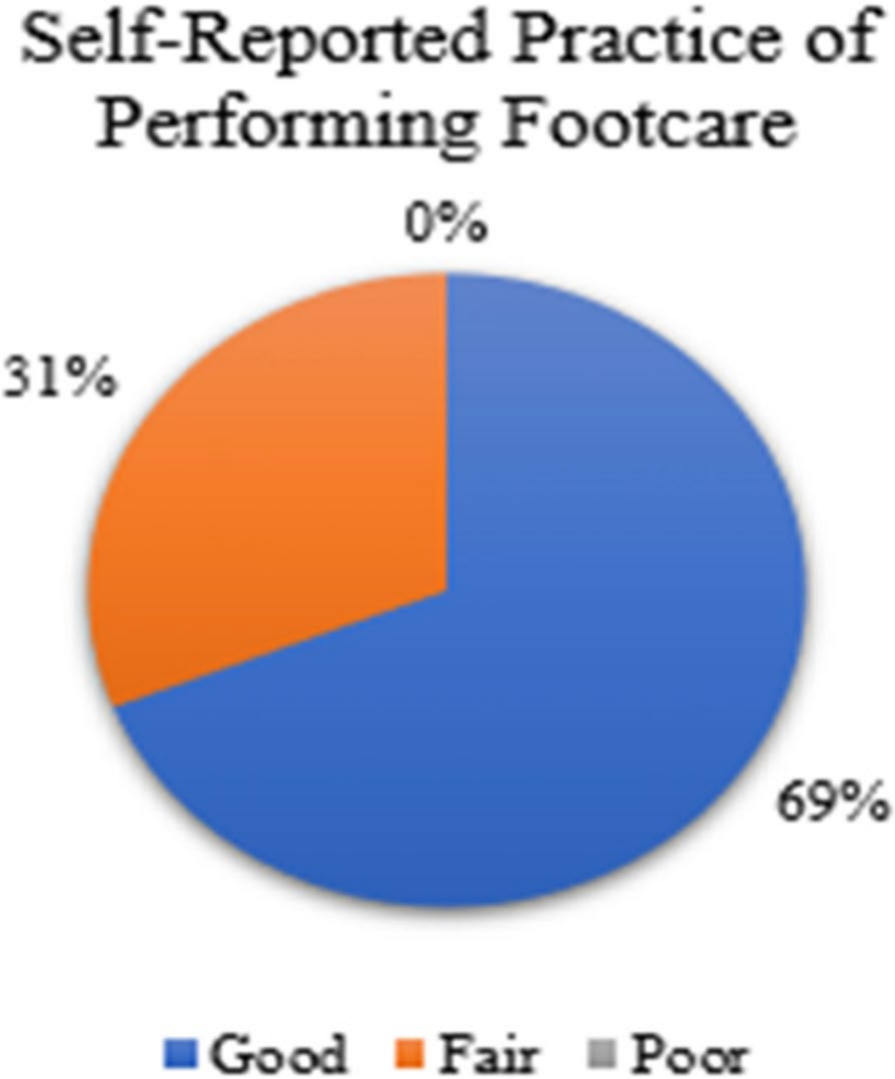
Distribution of the self-reported practice of performing footcare (*n* = 87)

With regard to research question 3, w*hat is the self-reported practice of persons with type II diabetes mellitus attending selected health centres in East Trinidad regarding; i. performing footcare, and ii. overcoming barriers to performing footcare?*, the scores of participants were used to determine the self-reported practice of performing footcare and overcoming barriers to performing footcare. The results are presented in Fig. 5. Summation of scores from section 4 of the questionnaire (self-reported practice of performing footcare) is illustrated in the Fig. 1. Most of the participants (69%) had good practice.

**Fig. 6:**
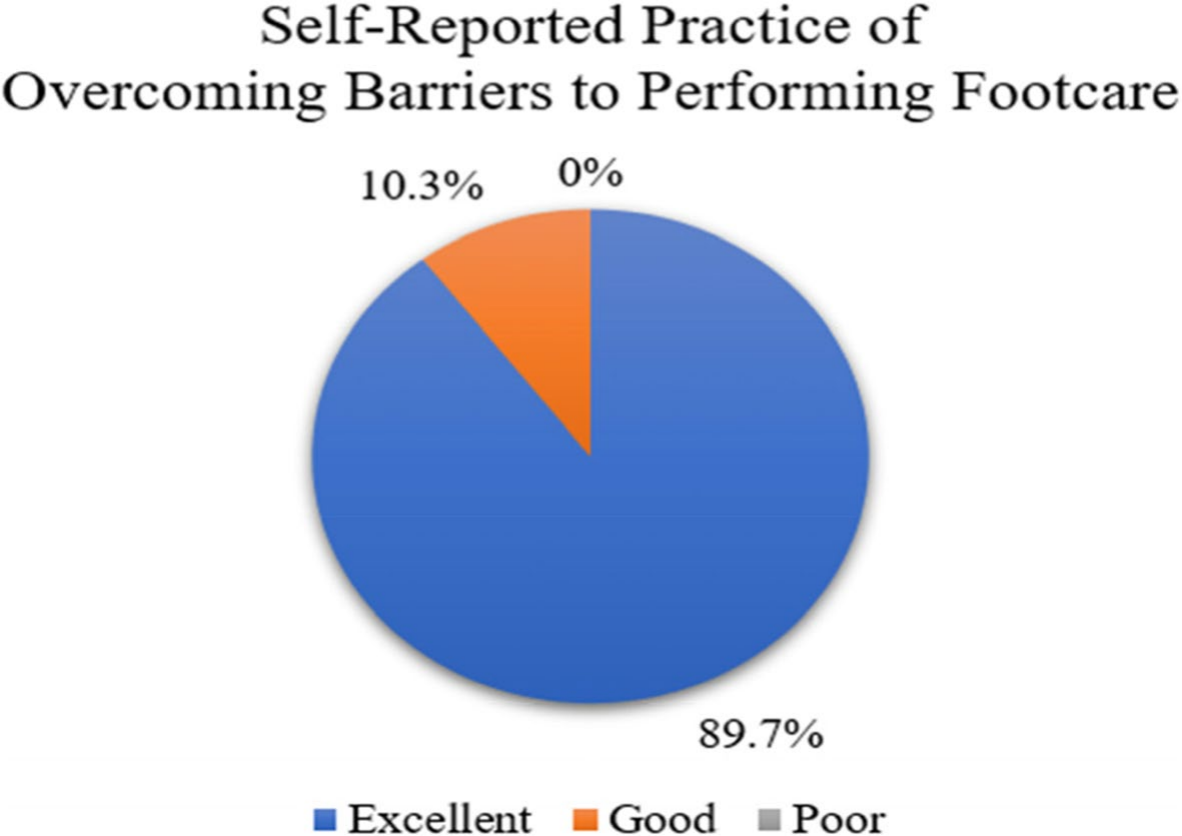
Distribution of the self-reported practice of overcoming barriers to performing footcare (*n* = 87)

The summation of scores from section 5 of the questionnaire (self-reported practice of overcoming barriers to performing footcare) are illustrated in Fig. 6. The figure illustrates that 89.7% of the participants reported ‘excellent practice’.

Table 3 is used to illustrate the responses of the respondents related to research question 4, a*re there any associations between; a. the sociodemographic characteristics and the beliefs of persons with type II diabetes mellitus attending selected health centres in East Trinidad, b. the sociodemographic characteristics and self-reported footcare practice of persons with type II diabetes mellitus attending selected health centres in East Trinidad?*

**Table 3:** Association between sociodemographics, footcare belief and self-reported footcare practice (*n* = 87)

		*Gender*	*Age*	*Ethnicity*	*Religion*	*Educational level*	*Employment status*	*Time since diagnosis*	*Health centre*
*Belief regarding susceptibility to a foot injury*	*Pearson correlation* *Sig. (2-tailed)*	*.133* *.219*	− .212^*^ *.049*	*− .064* *.557*	*.085* *.433*	*− .101* *.352*	*− .087* *.424*	*− .190* *.078*	*.003* *.977*
*Belief regarding the seriousness of complications*	*Pearson correlation* *Sig. (2-tailed)*	*.031* *.772*	*− .086* *.430*	*− .102* *.347*	*− .008* *.943*	*− .120* *.270*	*− .065* *.549*	*.008* *.940*	*.055* *.614*
*Self-reported Practice of performing footcare*	*Pearson correlation* *Sig. (2-tailed)*	*.012* *.910*	*− .160* *.138*	*.163* *.131*	*.093* *.391*	*− .036* *.744*	*− .122* *.260*	*− .188* *.082*	*.111* *.307*
*Self-reported practice of overcoming barriers to performing footcare*	*Pearson correlation* *Sig. (2-tailed)*	*.032* *.767*	*.016* *.882*	*− .019* *.859*	*− .073* *.501*	*.024* *.827*	*.125* *.250*	*.160* *.138*	*.181* *.093*

*Correlation is significant at the 0.05 level (2-tailed)

**Correlation is significant at the 0.01 level (2-tailed)

The table indicates that there is a statistically significant relationship between age of the participants and their beliefs regarding susceptibility to foot injury (*p* ≤ 0.05). There were no statistically significant relationships between their sociodemographic characteristics and their self-reported footcare practice of persons with type II diabetes mellitus attending selected health centres (*p* ≤ 0.05). The results are illustrated in Table 3.

Table 4 is used to respond to research question 5, a*re there any associations between the beliefs of the participants; i. related to their susceptibility to a foot injury, ii. regarding the seriousness of complications of injury to their foot, and the self-reported practice, (a) performing footcare and (b) overcoming barriers to performing footcare, of persons with type II diabetes mellitus attending selected health centres in East Trinidad?*

**Table 4:** Associations between footcare belief and self-reported footcare practice (*n* = 87)

		Self-reported practice of performing footcare	Self-reported practice of overcoming barriers to performing footcare
Belief regarding susceptibility to a foot injury	Pearson correlation Sig. (2-tailed)	.381^**^ .000	− .122 .259
Belief regarding the seriousness of complications	Pearson correlation Sig. (2-tailed)	.282^**^ .008	.251^*^ .019

*Correlation is significant at the 0.05 level (2-tailed)

**Correlation is significant at the 0.01 level (2-tailed)

The relationship between the belief regarding susceptibility to foot injury and the self-reported practice of performing footcare was statistically significant (*p* ≤ 0.05) and so was the relationship between the belief regarding the seriousness of complications and the self-reported practice of performing footcare was statistically significant (*p* ≤ 0.05). Similarly, we observed that the relationship between the belief regarding the seriousness of complications and the self-reported practice of overcoming barriers to performing footcare was statistically significant (*p* ≤ 0.05).

## Discussion

This study showed that the sociodemographic characteristics of the persons with type II diabetes mellitus attending *selected health centres* in East Trinidad is such that there were more females than males, the highest proportion of the study population was in the age group 49–65 years, of East Indian ethnicity, identified as being Christians, attained secondary education as the highest level of education, retired and diagnosed with type II diabetes mellitus for more than 5 years. This population represents a typical patients we attend to in the chronic disease clinics throughout the twin Island of Trinidad and Tobago. It is therefore pertinent that the findings could be a window to what is typical in the parent population.

Attention should be given to the beliefs regarding footcare and the practice of footcare of individuals with type II diabetes mellitus, to resolve the problem of the high incidence of foot ulcers leading to amputations in persons living with diabetes [32–34].

We note the finding that most of the participants had ‘strong’ beliefs regarding the susceptibility to a foot injury. Some of these beliefs regarding susceptibility to foot injury include but not limited to ‘diabetes can be dangerous to ones feet, even a small cut can have serious consequences,’ ‘diabetes may also reduce blood flow to the feet, making it harder to heal an injury and resist infection’ and ‘walking with bare feet increases the risk of injuring the feet.’ Similarly, in a recent study conducted in Tobago, most participants believed that walking barefooted exposes the foot to being pierced by any object, leading to a deep cut, foot ulcer, foot infection, and, consequently, amputation of the foot. This finding conform to Adeyemi et al. [35].

However, our finding that most of the participants had strong beliefs regarding the seriousness of complications is in contrast with some studies. Some of the beliefs regarding the seriousness of complications participants included ‘people with diabetes are far more likely to have a foot or leg amputated than other people,’ and ‘an amputation of a toe can become infected and subsequently lead to amputation of the limb.’ This finding contrasts with other studies that showed participants’ low perceived susceptibility and severity attributed to low perceived threats to the consequences of diabetes that shaped passive health-related attitudes [36–38].

The self-reported practice of performing footcare was mainly ‘good.’ Participants in this study reported frequent practice of washing feet daily with soap and water, inspecting feet for abnormalities, drying in between toes thoroughly, moisturizing feet but not in between toes, wearing soft, clean socks, cutting nails carefully, wearing proper fitting shoes and seeking medical attention for any injury to feet. Other studies found that most diabetic patients generally have a mixture of proper and improper footcare practices [7, 17]. Evidence shows that when individuals with diabetes believe the recommended self-care behaviours impinge their ability to maintain and enjoy ‘normal life,’ such belief can lead them to take the risk of ignoring the diabetes self-management recommendations, leaving wounds unattended and delaying seeking medical intervention which leads to amputation [36].

Although most participants were in the 49–65 years age group, the self-reported practice of overcoming barriers to performing footcare was ‘excellent.’ An older age is associated with a higher presence and increased severity of co-morbidities, which restrict the ability to perform self-care activities and are considered non-modifiable predisposing barriers to proper diabetic footcare [37]. To overcome barriers to performing footcare, participants in this study use mirrors to inspect below their feet, obtain professional service to maintain toenails and ask for assistance from a family member to inspect below their feet and trim their toenails. Thus, family support is a notable contribution to the maintenance of footcare practices in the elderly population of individuals living with diabetes mellitus. Thus, the statistically significant relationship between age and the belief regarding susceptibility to a foot injury (*p* ≤ 0.05) is noteworthy. Ageing brings changes to foot health and foot function with a greater prevalence of foot problems in later life [37]. Literature shows that attitudes about ageing influence psychological and health outcomes as an individual ages [17]. Apart from age, there was no other sociodemographic characteristic with a significant correlation to beliefs. Therefore, this study did not corroborate previous findings that a lower educational level, and lower socioeconomic status, had a higher presence of misperceptions [17].

The Health Belief Model is a health-specific behavioural cognitive model that was created to explain behaviours [36]. This study corroborates the correlation between belief and practice in remarkable agreement with the Health Belief Model. The results of the association between belief and the self-reported practice substantiate previous findings in the literature that indicate individuals will act to protect their health if there is a perceived threat to personal health and the conviction that the benefits of taking action to protect health outweigh the barriers that will be encountered [19].

Educating patients is likely to be effective if healthcare professionals are current with knowledge and practices on footcare [21]. Healthcare workers should not assume that an individual has counter-productive beliefs or improper footcare practices based on religious affiliation, educational level or economic status. Alternatively, individualized patient-centred health education should be employed as the beliefs and practices may vary for each person. It is essential to assess patients’ beliefs and behaviour to offer education and use educational methods that enable individuals with type II diabetes mellitus to care for their feet efficiently [21].

### Recommendations

This study can serve as a basis for further studies on footcare beliefs and practices as the results of this study should be validated by larger sample size. The prospect of reducing complications of injury to the feet of individuals with type II diabetes mellitus serves as an incentive for future research in this area. On a wider level, research is needed to determine approaches to support and maintain long-term behaviour change in foot self-care management of individuals living with diabetes. Additional investigation into the lived experience of individuals who experienced diabetic foot complications can be done to better understand the role of footcare beliefs and practices.

In addition to teaching about the pathophysiology of diabetes mellitus, and therapeutic nursing management, we recommend continued emphasis on diabetic footcare as part of primary prevention and health promotion, to respond to the global challenge of increasing incidence of diabetes mellitus [1 and 2]. Additional training or refreshment courses can be done via staff development units to equip nurse and other public health providers with the competencies required for assessment of patient’s current footcare practices, modification of beliefs toward footcare and promotion of proper footcare practices.

### Limitations

Gathering data via telephone interviews facilitated data collection with minimal risk of contributing to the spread of the COVID-19 virus during the pandemic. However, conducting telephone interviews was time-consuming, costly and presents a source of uncertainty as the practice of footcare of the respondents was not observed but self-reported. Due to the possibility that individuals could embellish the information stated, the findings are reliant on the honesty of the participants. The number of individuals scheduled for chronic disease clinics was reduced due to the COVID-19 pandemic. Some contact numbers given to the health centres were not answered to even with repeated trials or were not in service. All persons scheduled for the Chronic Disease Clinic at the selected health centres did not consent to participate in the study. Given that the findings from this study are based on a limited number of participants, we caution that about making generalized interpretations from the results. Future research may benefit from including people living in other geographical areas.

## Conclusion

This paper gives an account of the beliefs and self-reported practices of footcare of individuals with type II diabetes mellitus at selected health centres in East Trinidad. Evidence from this study supports the Health Belief Model as an effective framework for the promotion of appropriate footcare among persons with type II diabetes mellitus. Results indicated that persons with type II diabetes mellitus attending selected health centres in East Trinidad had strong belief regarding susceptibility to a foot injury, strong belief regarding the seriousness of complications of a foot injury, reported good footcare practice and excellent practice of overcoming barriers to performing footcare. There are associations between (a) the belief regarding susceptibility to a foot injury and age (*p* ≤ 0.05) and (b) between belief regarding susceptibility to a foot injury, seriousness of complications and self-reported footcare practices (*p* ≤ 0.05).

## Data Availability

The authors declare that all data and material in the study will be made available whenever they are requested.

## Abbreviations

IDF: International Diabetes Federation
HBM: Health Belief Model
